# Unbiased Phenotype Detection Using Negative Controls

**DOI:** 10.1177/2472555218818053

**Published:** 2019-01-07

**Authors:** Antje Janosch, Carolin Kaffka, Marc Bickle

**Affiliations:** 1 Technology Development Studio, Max Planck Institute of Molecular Cell Biology and Genetics, Dresden, Germany; 2 Fraunhofer-Institut für Verkehrs- und Infrastruktursysteme, Dresden, Germany

**Keywords:** high-content screening, multiparametric analysis, fingerprinting

## Abstract

Phenotypic screens using automated microscopy allow comprehensive measurement of the effects of compounds on cells due to the number of markers that can be scored and the richness of the parameters that can be extracted. The high dimensionality of the data is both a rich source of information and a source of noise that might hide information. Many methods have been proposed to deal with this complex data in order to reduce the complexity and identify interesting phenotypes. Nevertheless, the majority of laboratories still only use one or two parameters in their analysis, likely due to the computational challenges of carrying out a more sophisticated analysis. Here, we present a novel method that allows discovering new, previously unknown phenotypes based on negative controls only. The method is compared with L1-norm regularization, a standard method to obtain a sparse matrix. The analytical pipeline is implemented in the open-source software KNIME, allowing the implementation of the method in many laboratories, even ones without advanced computing knowledge.

## 陰性対照群を用いた偏りのない表現型の検出

**抄録**

自動顕微鏡検査を用いた表現型のスクリーニングでは、スコアリング可能なマーカー数や抽出可能なパラメータの豊富さによって、化合物が細胞に及ぼす影響を包括的に計測することが可能である。高次元のデータは豊富な情報源であると同時に、情報が隠される可能性があるノイズの源でもある。その複雑さを抑え、興味深い表現型を特定するために、こうした複雑なデータを扱う方法が数多く提案されてきた。それにもかかわらず、大半の研究所では解析の際に1つか2つのパラメータのみを依然として用いているが、これはより高度な解析を行う計算上の課題による可能性が高い。本稿でわれわれは、これまで知られていなかった表現型を陰性対照群のみに基づいて発見することを可能にする新たな方法を提示する。その方法は、疎行列を生成する標準的な方法であるL1ノルム最小化と比較される。オープンソースソフトウェアのKNIMEでは解析パイプラインが実装されており、このKNIMEによって、コンピュータに関する高度な知識を持たないところもある研究所の多くでこの方法の実施が可能になっている。

## 음성 통제를 활용한 비편향 표현형 검출

**초록**

자동현미경을 활용한 표현형 선별검사는 점수화할 수 있는 표지자 수 및 추출할 수 있는 모수에 따라 화합물의 세포에 미치는 영향을 종합적으로 측정한다. 데이터의 고차원은 풍부한 정보원임과 동시에 정보를 감출 수 있는 잡음원이기도 하다. 이러한 복잡한 데이터를 다루어 복잡성을 줄이고 흥미로운 표현형을 파악하기 위해 다양한 방법들이 제시되었다. 하지만 실험실 대다수에서 정교한 분석의 컴퓨팅이 어렵기 때문에 분석에 1~2가지의 모수만을 사용하는 실정이다. 본 논문에서는 이전에는 알려지지 않은 음성 통제 기반 표현형을 발견하는 새로운 방법을 제시하였다. 본 방법을 희소 행렬을 확보하는 표준 방법인 L1-정규화와 비교하였다. 분석 파이프라인을 고급 컴퓨팅 지식이 없어도 많은 실험실에서 구현할 수 있는 방법인 오픈 소스 프로그램 KNIME에서 구현하였다.

## 使用阴性对照进行无偏倚的表型检测

**摘要**

使用自动显微镜进行的表型筛选凭借可评分标记的数量以及可提取参数的丰富性，能够全面地测量化合物对细胞的作用。数据的高维度既是丰富的信息来源，也是可能掩盖信息的噪声来源。已有许多处理这些复杂数据的方法被提出，从而降低复杂性并识别相关表型。然而，大多数实验室在分析中仍仅使用一个或两个参数，这可能是由于进行更复杂的分析所面临的运算挑战。在本文中，我们提出了一种新型的方法，其能够仅基于阴性对照就能发现新的、之前未知的表型。我们将这种方法与一种获得稀疏矩阵的标准方法——L1范数正则化——进行比较。分析流程在开源软件KNIME中实现，从而让许多实验室都能实现这一方法，即便是没有高级运算知识的实验室。

## Introduction

Of the many ways to unlock the mechanisms by which cells respond to their environment, high-content screening has established itself as a powerful technology. Several reasons can be invoked for this popularity, the major one being the richness of information that can be obtained from images. The richness comes in many layers. First, several organelles or proteins can be marked simultaneously, allowing the gathering of information about several concurrent biological effects. Second, for each labeled cellular component many parameters can be extracted that not only measure its amount but also its morphology and its distribution in the cell with respect to itself and other structures in the cell. Lastly, this analysis can be carried out on a cell-by-cell basis, giving insight into population distribution instead of simply its average. Few other methods yield such diverse information about cells.

The richness of the information extracted is also a challenge, both mathematically and computationally. Depending on the resolution of the images, between 200 and 2000 cells are commonly imaged per condition in a screen. A screen with 100,000 data points and 1000 parameters yields a matrix of 2 × 10^8^ rows and 1 × 10^3^ columns whose size can be nearly as large as the original image data (no compression). The data are also complex in that they represent different measurements with different units, some of which are correlated with others, some of which are not informative and add noise without helping to discriminate the signal, and lastly, some that are noisy and tend to mask the signal. A further complication is that some parameters do not help the biologist understand what the phenotype actually looks like and its biological significance. This is particularly true for texture features that are hard to interpret in a biologically meaningful manner. Since data mining is the task of transforming data into knowledge or insights, some parameters need to be ignored when interpreting phenotypes.

The challenging nature of data mining is reflected in the fact that most high-content screening publications to date make use of only a few parameters instead of delving into the richness of the data they have obtained.^1^ Obviously, to discover hits in a very focused assay, only a few parameters are required. Nevertheless, when more parameters are exploited, the hits can be profiled according to their phenotypes, allowing a stratification strategy for selecting compounds for further development.^2^ Furthermore, if the assay has been well characterized with a set of compounds with a known mode of action, the hits can potentially already be classified with respect to their mode of action, allowing quick identification of compounds acting via novel mechanisms.

Several strategies have been developed to use the full range of parameters extracted from the images, each with their own advantages and disadvantages. These can be broadly split into supervised methods and unsupervised methods. Supervised methods use various machine learning algorithms (support vector machines,^3^ random forests,^4^ deep neural networks,^5^ bagging,^6^ etc.) to classify cells into phenotype categories defined with a training set. These methods use many parameters and are very powerful and relatively easy to use. Their main disadvantage is that they have to classify objects into one of the user-defined categories, so that novel, previously unseen phenotypes will be wrongly classified (open world vs closed world).^7^ For example, training a machine learning algorithm on cat images will wrongly classify a dog as a breed of cat. This problem is not addressed in the field, with a few exceptions.^8^ A further problem of supervised methods is the danger of overfitting or training the algorithm on some latent feature.^9^ Overfitting leads to overestimation of the efficacy of the algorithm and poor performance on real, unknown data. Training on latent features can lead the algorithm to recognize features outside of the objects of interest. For instance, if the controls used for training are always in the same position, the algorithm might recognize the position due to peculiarities in the way the microscope images those positions.

Several unsupervised methods have been proposed; in particular, two publications did a systematic review and tested many of these.^10,11^ In both papers, the task was to evaluate the ability of various dimension reduction methods and, for Reisen et al., evaluate various distance measures to classify or score a high-dimensional phenotypic dataset. Reisen et al. demonstrated that the full-length phenotypic readout distinguishes less well between a positive and a negative control than the best single parameter, demonstrating the need to perform dimension reduction. They then demonstrated that single parameters are very poor at profiling compounds and finding compounds of a similar mode of action. Instead, the full-length fingerprint appears to recognize similar phenotypes slightly better than reduced dimensionality data by principal component analysis (PCA) or random forest scales (RFSs). Ljosa et al. demonstrated that for profiling compounds, their full fingerprint performs very well and is not outperformed by methods meant to capture the population response. In their analysis, dimension reduction by factor analysis does significantly improve the classification of the compounds. This demonstrates that some parameters in their fingerprint are noisy and affect the classification.

We sought to develop a profiling method that is able to discover previously unseen phenotypes, exploit the population response, and avoid noisy or uninformative parameters. We reasoned that if we obtain the set of parameters that describes the negative population precisely and reproducibly, we would reduce the dimensionality without sacrificing the ability to discover new phenotypes, while eliminating noisy, unreliable parameters. For instance, if the area of nuclei is always to be found within a given range in all the wells of the profiling experiment, then any condition where the area diverges significantly is a phenotype. Conversely, if another parameter exhibits diverging ranges in different wells, this would not be reliable and the parameter should be eliminated. To achieve this, our method analyzes the distribution of populations in all wells for each parameter available and selects parameters that are neither too noisy nor too uninformative. We used the publicly available data of BBBC021 and BBBC022 from the Broad Institute to test our novel method and demonstrate its performance. We compared our method for reducing parameter space using negative controls only with the classic method of L1-norm regularization.^12^ We found that our method improves the classification performance compared with the full matrix, but it does not outperform the sparse matrix obtained by L1-norm. We then showed that our method generalizes better when an unseen class is added to the classification problem compared with the sparse matrix obtained by L1-norm.

## Materials and Methods

### Data

We used image set BBBC021v1,^13^ available from the Broad Bioimage Benchmark Collection.^14^ The BBBC021 dataset is of Mcf7 breast cancer cells that were plated and treated with compounds of a known mode of action.^13^ The images were reanalyzed with CellProfiler by Ljosa et al.^11^ The resulting data were made publicly available and are available on the Broad Institute’s website. The data were imported into a mysql database as described by Ljosa et al. and imported into KNIME with corresponding database reader nodes. The data were then normalized per plate as described in by Ljosa et al. by linearly scaling the data, setting the 1st percentile of DMSO to 0 and the 99th percentile to 1.

We used image set BBBC022v1,^15^ available from the Broad Bioimage Benchmark Collection.^14^ The BBBC022 dataset comprises U2OS cells treated with 1600 known bioactive compounds. The cells were labeled with six dyes to characterize seven organelles (Cell Painting Assay).^15^ We downloaded the images from the Broad website (https://data.broadinstitute.org/bbbc/BBBC022/) and analyzed them with the CellProfiler pipeline provided by Gustafsdottir et al. without image correction. The fingerprints were annotated with the mode of action provided in Rohban et al.^16^ The BBBC022 data were not scaled, but the well averages were z-normalized per plate with respect to the DMSO controls. The BBBC021 dataset consists of three replicates, whereas the BBBC022 dataset consists of replicates. Either the replicates were analyzed separately or the median of the replicates was calculated.

### Binning Analysis

For subpopulation analysis, we applied the binning analysis that was previously described.^17^ Briefly, the DMSO population for each parameter was sorted from the smallest to the biggest value and the cutoff values at the 20th, 40th, 60th, 80th, and 100th percentiles were determined, creating five bins. Each DMSO well then had five bins per parameter, all containing approximately 20% of the population. The distribution of the counts was approximately normal, allowing us to compute a z score for the counts in each bin and each parameter of each experimental well. The binning analysis was carried out per plate with the DMSO population of the entire plate.

### Stability of Parameter Analysis

The range of each 20% bin of each parameter was determined over the entire DMSO population of a plate. For each well of the plate, it was then expected that 20% of the population should be found in each bin for each parameter. The actual counts of cells per well and parameter followed a multinomial distribution since the procedure is similar to drawing a ball from an urn with replacement. Therefore, to test whether the obtained distribution of counts for each parameter and each well corresponded to a 20%, 20%, 20%, 20%, 20% distribution, a chi-square test was applied. The resulting *p* value was adjusted for multiple testing with the Bonferroni method.

### L1 Regularization

L1 regularization was implemented in the KNIME workflow in a Python scripting node. LogisticRegression from the sklearn library was used, and the tuning parameter alpha was set at 0.01, 0.05, 0.1, 0.5, 1, 2, 4, 6, 8, and 10. At each alpha, some parameters had 0 coefficients and fingerprints were constructed without these parameters and used for classification.

### Classification

The distance matrix of the profiles was calculated with cosine as the distance measure. The nearest-neighbor algorithm with leave-one-out cross-validation was applied to determine the most similar compound and its mode of action. The number of correctly classified compounds was reported.

### Software

The workflows were implemented in KNIME. The normalization script was written in Python and a graphical user interface (GUI) was generated for KNIME with the RGG interface. The chi-square test was carried out with R from within the KNIME workflow using a R snippet node. All workflows and instructions for installing KNIME with extensions are available for download at https://publications.mpi-cbg.de/publications-sites/7217/.

## Results

### Fingerprint Profiling with Well Averages

We wished to test whether analyzing subpopulations and reducing the parameter space by selecting parameters that describe a negative control population robustly could improve phenotypic fingerprint classification tasks. To this end, we built an analysis pipeline based on the benchmarking datasets BBBC021 and BBBC022 of the Broad Institute. For the BBBC021 dataset, we downloaded the fingerprints, and for the BBBC022 dataset, we downloaded the images and performed image analysis with the CellProfiler pipeline provided with the dataset. We thereby obtained two annotated datasets of individual cells treated with compounds and described by many quantitative parameters. The BBBC021 dataset has 454,695 cells treated with 96 compounds in triplicate and 7 compounds in duplicate described by 460 parameters. Only a subset of the BBBC022 dataset was used in this analysis, representing 2,894,683 cells treated with 591 compounds classified in 59 modes of action.

First, we tested the simplest analysis possible for classifying fingerprints by calculating the arithmetic mean of the single-cell data of each well. We classified both the individual profiles and the median of the replicates of both datasets. For classification, we found the mode of action of the most similar compound based on its cosine distance. We found that for the BBBC021 dataset, 289 out of 302 (94.7%) individual fingerprints were correctly classified (**Table 1**). The BBBC022 dataset showed poorer classification, with 419 out of 2372 individual fingerprints (17.7%) correctly classified. In summary, the BBBC021 dataset can be classified with very high accuracy using the average of the cell data, whereas the BBBC022 dataset shows much more modest accuracy.

**Table 1.** Classification Accuracy of the Various Methods with the BBBC021 and BBBC022 Datasets.

	BBBC021			BBBC022		
Method	% Correct MoA	Correct MOA	Wrong MOA	% Correct MoA	Correct MOA	Wrong MOA
Well averages	95.70%	289	13	17.66%	419	1953
Binned parameters	94.70%	286	16	19.39%	460	1912
Binned parameters, 20% and 100% bins	94.04%	284	18	19.65%	466	1906
Well averages, L1-norm regularization	92.05%	278	24	20.53%	487	1885
Binned parameters, L1-norm regularization	95.03%	287	15	19.56%	464	1908
Binned stable parameters	95.03%	287	15	19.22%	456	1916
Binned stable parameters, 20% and 100% bins	93.38%	282	20	20.19%	479	1893

MoA = mode of action.

### Fingerprint Profiling with Subpopulation

**Figure 1.**
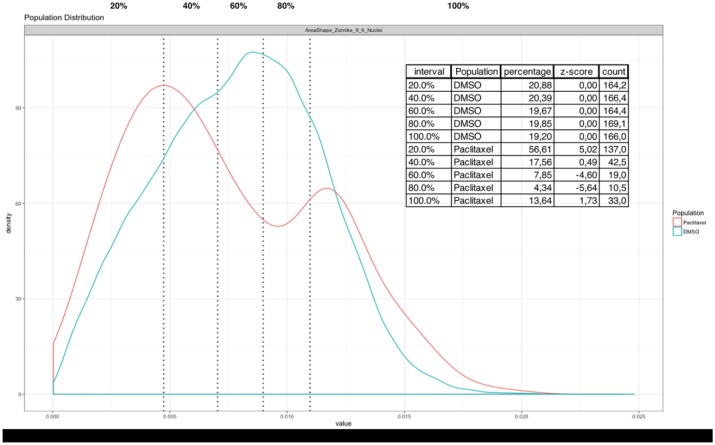
Distribution of the cell populations of DMSO and paclitaxel in plate 20585 of the BBBC022 dataset for the eighth order of the Zernike polynomial at a scale of 6 pixels of the nuclei. The dotted lines represent the separation between the 20th, 40th, 60th, and 80th percentiles. Paclitaxel transforms a unimodal distribution to a bimodal distribution, resulting in an increased number of objects in the low 20% and 100% bins. The inset shows the percentage, z score, and count for each bin for the DMSO population of the plate and the paclitaxel well.

After establishing a baseline performance for profiling compounds using population means, we wanted to investigate whether incorporating subpopulation information could be beneficial for the accuracy of the classification. We have previously developed a method for subpopulation analysis called binning analysis.^17^ Briefly, users define how many equally sized bins into which to split the populations in the wells. For five bins, the 20th, 40th, 60th, and 80th percentiles are determined for the control population for each parameter. Control populations can be all the negative wells in a plate, or in batches of plates or an entire screen. Each bin therefore determines a parameter range for each percentile. The limits of the ranges are then applied to experimental wells to determine the number of cells in each bin. For each well, each parameter, and each bin, the output of our method yields the population percentage, the counts, and the z scores based on the mean and standard deviation of the percentages of the negative control population. Phenotypic changes result in a shift of the population distribution among the bins compared with the negative control. To illustrate the behavior, we show for the BBBC022 dataset the eighth order of the Zernike polynomial at a scale of 2 pixels for DMSO and paclitaxel with five bins. The population distribution of paclitaxel is shifted toward the higher (100%) and lower (20%) bins (**Fig. 1**). Averaging such a parameter for the well population yields a z score of −3, whereas the binning analysis yields a z score of 6.7 for the 20% bin, 2 for the 40% bin, 6.6 for the 60% bin, −9.1 for the 80% bin, and 4.7 for the 100% bin. This method therefore allows us to detect weak phenotypes and explains the population distribution inherently.

Here, we carried out the binning with five bins using the DMSO population distribution per plate. To estimate the effect of binning on the ability to distinguish the various modes of action present in the dataset, we again performed nearest-neighbor classification. Since for some parameters not all bins are instantiated, we first remove parameters with missing values in order to calculate the cosine distance matrix used in nearest-neighbor classification. Binning the BBC021 dataset resulted in a vector with 2247 parameters for the individual replicates. With the binned data, we correctly classified 286 out of 302 compounds (94.7%) (**Table 1**). The subpopulation analysis of the binned profiles correctly classified one compound less than the mean profiles. We reasoned that the accuracy could be improved just by carrying out the classification using either the lowest-range bin (first 0%–20% bin) or the largest-range bin (80%–100% bin), since changes in phenotypes will be reflected by parameters becoming either smaller or greater, and thus only the extreme bins will be affected. Carrying out nearest-neighbor classification with the 100% and 20% bins decreased the classification performance to 284 out of 302 replicates (94.0%). The same analysis for the BBBC022 dataset yielded a classification with all bins of 460 correctly classified profiles (19.4%), compared with 419 with well population average. Using only the 100% and 20% bins, we were able to further improve the classification to 466 correctly classified profiles (19.7%). In summary, classifying compounds with the 100% and 20% bin profiles significantly improved the classification accuracy for the BBBC022 dataset, but slightly decreased the classification accuracy for the BBBC021 dataset.

### L1-Norm Regularization for Parameter Reduction

A disadvantage of our binning analysis is that the number of parameters increases, which worsens the dimensionality problem for distinguishing phenotypes. We therefore wanted to reduce the number of parameters and obtain a sparse matrix capable of distinguishing better phenotypes. A popular approach to reduce the parameter space when a ground truth is known is L1-norm regularization.^12^ The ground truth is used to carry multiple logistic regression, and L1-norm regularization sets parameters to 0. A tuning factor alpha is scanned with values between 0.01 and 10, with 10 different values yielding 10 fingerprints with different selections of parameters. The classification task was carried out with each fingerprint and the accuracy assessed. We first applied L1-norm regularization to both the well-averaged populations and the binned population. The accuracy of the classification with L1-norm regularization for the well-averaged populations of the BBBC021 dataset was decreased, with 278 compounds correctly classified, compared with 289 for the entire parameter set. L1-norm regularization modestly improved the classification of the binned individual replicates to 287 correct classifications, compared with 286 for all the bins. For the well-averaged BBBC022 dataset, L1-norm regularization increased the classification accuracy to 487 (20.5%) profiles compared with 419 for the well averages. L1-norm regularization did not improve the classification of the binned data, with 464 (19.7%) of correctly classified profiles compared with 466 profiles with the high and low bin profiles only.

### Removal of Noisy Parameters

**Figure 2.**
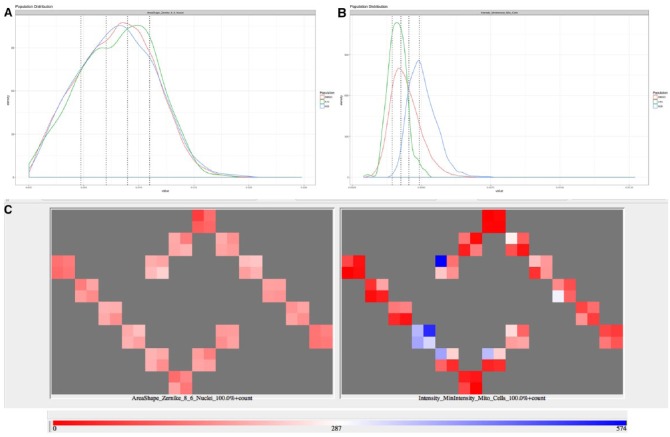
(**A**) Population distribution of a stable parameter (eighth order of the Zernike polynomial at a scale of 6 pixels) for the entire DMSO population of a plate (red) and two DMSO wells from the same plate (green, blue). (**B**) Population distribution of a noisy parameter (minimal intensity of Mitotracker in cells) for the entire DMSO population of a plate (red) and two DMSO wells from the same plate (green, blue). (**C**) Plate heatmap of the number of cells in the 100% bin for the DMSO wells of a single plate for a stable parameter (eighth order of the Zernike polynomial at a scale of 6 pixels; left) and a noisy parameter (minimal intensity of Mitotracker in cells; right).

Obtaining a sparse matrix with L1-norm regularization biases the model toward detecting phenotypes that are present in the training set. The optimized parameter set might not generalize well and might be unable to correctly classify unseen phenotypes. To avoid the problem of overfitting, we focused on selecting parameters only based on negative controls. We reasoned that parameters that describe the negative population faithfully and reproducibly should be sensitive to detecting deviations from the unperturbed state. We therefore analyzed the distribution of counts for each parameter in each well to assess whether it followed the expected distribution of the 20th percentile of the population in each bin. The distribution of the counts per bin is a multinomial distribution, and a chi-square test yields a *p* value for the likelihood that the observed counts belong to the expected multinomial distribution. The *p* value was then adjusted for multiple testing and a cutoff value of 0.05 was chosen to classify whether the counts obtained for a parameter in a well follow the expected multinomial distribution. For some parameters, only few wells showed the expected distribution, which we call noisy parameters, whereas for other parameters all wells were within the expected distribution, and we call these parameters stable (**Fig. 2**). We then built phenotypic fingerprints removing stepwise noisy parameters, obtaining fingerprints with more and more stable parameters (above the 0.05 cutoff). To build the fingerprints, we first removed parameters where only 1% of DMSO wells were above the 0.05 cutoff, then 6%, and so forth, until 96% of the DMSO wells were above the 0.05 cutoff in steps of 5%. We then performed the classification task with the 20 resulting fingerprints. For the BBBC021 dataset, we found that 287 compounds were correctly classified (95.0%) with the all bins, and using only the 100% and 20% bins, 282 compounds were correctly assigned their mode of action (93.4%). For the BBBC022 datasets, 456 (19.2%) and 479 (20.2%) compounds were correctly classified with all the bins or the 100% and 20% bins only, respectively. In summary, selecting stable parameters increased the classification accuracy compared with all binned parameters. Selecting parameters by L1-norm regularization was in most cases more powerful, though.

### Generalization of the Selected Parameters

Our rationale to select parameters based only on the description of the negative control was that we were not biasing the model toward known modes of action, whereas L1-norm regularization optimizes the model on the known modes of action. To directly test whether our method generalizes better, we retrained the models omitting a mode of action with many compounds. We chose dopamine receptor antagonist with 180 compounds and glucocorticoid receptor agonist with 140 compounds. We trained the L1-norm regularization model and selected the best threshold for stable parameters without one of these modes of action and verified the classification accuracy after adding the compounds and their mode of action back in the test set. Thus, the models were trained without seeing a class, which was then later added. We found that for the dopamine receptor antagonist mode of action, 118 compounds were not correctly classified with L1-norm regularization, whereas 115 compounds were not correctly classified with the stable parameter selection for either all bins or just the 20% and 100% bins. For the glucocorticoid receptor agonist mode of action, we found that 45 compounds were misclassified by L1-norm regularization compared with 49 and 37 for the stable parameter selection model. These results suggest that our stable parameter selection method with the 20% and 100% bins only allows building models that generalize better than L1-norm regularization.

## Discussion

We have taken advantage of two publicly available annotated high-content screening datasets to explore fingerprint analysis methods. We have compared a simple averaging of the well populations to subpopulation analysis and have compared the effect of dimensionality reduction on the classification task. The subpopulation analysis is based on the user-defined percentiles of the negative control population determining their parameter range. These parameter ranges are then used to determine the count of cells in each well. The number of bins in the analysis is important. At one extreme, having a single bin reduces the analysis to a simple cell count. At the other extreme, using too many bins, some bins will be empty or have only very few objects. It is therefore important to strike a balance between a high-resolution analysis of the data and no resolution at all. Additionally, some parameters cannot be binned, as they have too few values or a vast majority of the population have only one of the values. For instance, the number of nuclei per cell is often one. A binning of five for the number of nuclei per cell will not succeed, since a vast majority of cells will have a value of one and only a very few multinucleate cells will not be sufficient to create several 20% bins. In such a case, only the low-interval bin (20%) will be instantiated.

In our binning analysis, phenotypes causing small changes in parameters shifted the distribution of the population away from the user-defined percentiles. The significance of the distribution changes can be tested with a chi-square test. This method has the advantage that it handles various distribution types, such as bimodal or normal, equally well. One disadvantage is that statistical tests are sensitive to the number of objects, as the power of the test increases with larger populations. With small populations of a few hundred objects, large deviations from the expected quantiles are tolerated by the chi-square test, whereas only small deviations are tolerated with populations of several thousand cells. Due to this caveat, our method to detect noisy parameters for dimensionality reduction needs to be optimized for each dataset. Noisy parameters are determined by analyzing the negative controls only, thereby avoiding biasing the selection of parameters toward known compounds. The estimation of noisiness of a parameter is carried out by testing how many wells of the negative control population do not show the expected quantiles. We show that removal of the noisiest parameters does improve the classification accuracy substantially, although the best accuracy in the BBBC021 and BBBC022 is not achieved by this method. For the BBBC021, the best accuracy is achieved by well average, which is the simplest method. It must be noted that the BBBC021 dataset is very biased, containing very well-behaved experimental wells handpicked out of a larger dataset, and therefore achieves very high accuracy. Such accuracy is unlikely to be found in other datasets. For the BBBC022, the best accuracy is achieved with L1-norm regularization of the averaged population. For both the BBBC021 and the BBBC022 datasets, our method with binned, stable parameters is the second best. Given the good performance of our method in these two examples, it is likely that it will also perform well with other datasets. Generalization of the model obtained is one of the interesting features of our method. We show that selecting stable parameters with an unseen mode of action and then classifying the data with a new class leads to better accuracy than L1-norm regularization. This suggests that it is possible to train a model and reuse it even though the number and quality of classes have changed. Thus, when a researcher finds that the annotation of a compound has changed or been determined, all the compounds can be reclassified without first selecting stable parameters again.

Our analysis method can also be applied for tasks unrelated to fingerprint analysis. In our experience, the subpopulation analysis allows the detection of phenotypes where only a minority of cells show a phenotype. Our KNIME node calculates a z score for each parameter and each bin, based on the mean and standard deviation of the percentages in the DMSO wells. These z scores are used to detect phenotypes in screens, and we have detected weak, reproducible hits that were not detectable by population averages.

Our methodology could be further improved with more informative fingerprints. Here, we used only low-level features extracted from segmented images. Another approach could consist of applying machine learning such as deep learning to combine low-level parameters, either directly from the pixel intensities or from segmentation, and learn the relevant features.^18^ Such fingerprints containing high-level features might perform much better than the low-level features we used here.

We created our analysis in KNIME and made it publicly available with the analyzed data. Thanks to the intuitive user interface, non-computer scientists will be able to utilize the method for their own work. We hope that the community will take advantage of the method and provide feedback to further improve its performance.
